# Distinct Roles of Zmynd17 and PGC1α in Mitochondrial Quality Control and Biogenesis in Skeletal Muscle

**DOI:** 10.3389/fcell.2019.00330

**Published:** 2019-12-10

**Authors:** Kiyoshi Yoshioka, Ryo Fujita, Daiki Seko, Takashi Suematsu, Shinji Miura, Yusuke Ono

**Affiliations:** ^1^Department of Muscle Development and Regeneration, Institute of Molecular Embryology and Genetics, Kumamoto University, Kumamoto, Japan; ^2^Nagasaki University Graduate School of Biomedical Sciences, Nagasaki, Japan; ^3^Research Fellow of Japan Society for the Promotion of Science, Tokyo, Japan; ^4^Department of Human Genetics, McGill University, Montreal, QC, Canada; ^5^Division of Biological Macromolecular Research Support, Nagasaki University School of Medicine, Nagasaki, Japan; ^6^Laboratory of Nutritional Biochemistry, Graduate School of Nutritional and Environmental Sciences, University of Shizuoka, Shizuoka, Japan; ^7^Center for Metabolic Regulation of Healthy Aging, Kumamoto University Faculty of Life Sciences, Kumamoto, Japan

**Keywords:** skeletal muscle, mitochondria, Zmynd17, Mss51, Pgc1, glucose intolerance, metabolic homeostasis

## Abstract

Maintaining skeletal muscle mitochondrial quality is important not only for muscle activity but also for systemic metabolism. Exercise has long been recognized to have a positive impact on muscle mitochondrial quality. Although exercise triggers various changes in the mitochondrial dynamics, its molecular basis remains to be elucidated. We have previously reported that inactivation of the muscle-specific protein, zinc finger MYND domain-containing protein 17 (Zmynd17), results in mitochondrial abnormalities. To investigate the link between Zmynd17 activity and exercise-induced mitochondrial maintenance, we observed the effect of consecutive exercise on the mitochondrial quality in Zmynd17-deficient muscles. Zmynd17-deficient mice displayed abnormal mitochondrial morphology in limb muscles, which remarkably improved upon voluntary exercise. Interestingly, morphological abnormalities in mitochondria were even more apparent when PGC1α, a regulator of exercise-induced mitochondrial biogenesis, was overexpressed in Zmynd17-KO limb muscle. These abnormalities were also ameliorated by voluntary exercise. Our results show that neither the effect of consecutive exercise on mitochondrial quality nor PGC1α-induced mitochondrial biogenesis are mediated through Zmynd17 activity, thereby suggesting the existence of a complex mechanism of mitochondrial quality control in muscles.

## Introduction

Skeletal muscle contributes to approximately 40% of body mass in humans and plays a major role in systemic metabolism. Disruption of the muscle metabolic state often results in the development of chronic diseases, such as type 2 diabetes mellitus (Shigenaga et al., 1994; Navarro and Boveris, 2007; DeFronzo and Tripathy, 2009; Hesselink et al., 2016). Although mitochondria are central to the energy metabolism in skeletal muscle (Gerhart-Hines et al., 2007; Koves et al., 2008; Russell et al., 2014), the molecular mechanisms of mitochondrial quality control remain unclear. Recent studies show that exercise improves mitochondrial quality and function by stimulating their turnover (Safdar et al., 2011a; Cartee et al., 2016; Joseph et al., 2016). Peroxisome proliferator-activated receptor coactivator 1 alpha (PGC1α), a key regulator of mitochondrial biogenesis, is known to be upregulated by exercise training (Wu et al., 1999; Safdar et al., 2011b; Schnyder and Handschin, 2015; Narkar, 2017). Consecutive exercise training promotes degradation of abnormal mitochondria by autophagy, known as mitophagy (Vainshtein et al., 2015; Laker et al., 2017). Morphology and function of mitochondria are also regulated by fusion and fission, which are mitochondrial dynamics linked to exercise (Cartoni et al., 2005; Ding et al., 2010). Exercise is therefore a promising intervention for the treatment of metabolic diseases, including type 2 diabetes. Although the beneficial effects of exercise on muscle mitochondria are well-appreciated, the molecular link between exercise and mitochondrial quality control remains to be elucidated.

We have previously reported that the muscle-specific gene zinc finger MYND domain-containing protein 17 (Zmynd17) controls mitochondrial quality in muscle, especially in fast-glycolytic muscles (Fujita et al., 2018). *Zmynd17* deletion resulted in abnormal mitochondria in skeletal muscle. With aging or under metabolic stress induced by a high-fat-diet, Zmynd17-deficient (*Zmynd17*-KO) mice exhibited exacerbated muscle mitochondrial morphology in skeletal muscle, along with glucose intolerance. However, how regular exercise is related to the protective role of Zmynd17 in muscle mitochondria is unclear. Here, we used *Zmynd17*-KO mice and muscle-specific PGC1α transgenic mice to investigate the mitochondrial-quality regulation in the context of voluntary exercise. To elucidate the link between Zmynd17 activity and exercise induced mitochondrial maintenance, we examined the effect of voluntary exercise on the mitochondrial quality in Zmynd17-deficient muscles.

## Materials and Methods

### Animals

Animals were handled according to the approved protocols and guidelines of the Animal Committee of Nagasaki University. Mice were allowed *ad libitum* access to water, standard rodent chow (CE-2, CLEA Japan, Tokyo, Japan). *Zmynd17*^*LacZ/LacZ*^ homozygous [knockout (KO)] mice were generated as previously described. C57BL/6 *Zmynd17*^*LacZ/+*^ embryonic stem (ES) cells (Clone: 14311E-G5) were obtained from the Knockout Mouse Project repository (University of California, Davis, Davis, CA, United States). *Zmynd17*-KO mice and littermate control wild-type (WT) mice were analyzed at the age of 4–5 months, unless otherwise indicated. Male mice were used in all experiments. Transgenic mice overexpressing PGC1α in skeletal muscle (PTG mice) were generated as previously described (Miura et al., 2006) and crossed with *Zmynd17*-KO mice to generate *Zmynd17^*LacZ/LacZ*^;PGC1*α*-transgenic* (KO-PTG) mice. Mice assigned to exercise group were individually housed in cages equipped with running wheel (RW-15, Melquest, Toyama, Japan). Daily running distance was approximately 10 km in the exercise group (data not shown).

### Grip Strength Test

Forelimb grip strength was measured, using a Grip Strength Meter (Columbus Instruments, Columbus, OH, United States) for mice, as previously described with some minor modifications (Fujita et al., 2018). Peak tension [in neuton (N)] was recorded when the mouse released its grip. Three sets of five successive measurements were performed for each mouse. The peak value was defined as mouse fore-limb grip strength.

### Running Performance Test

Mice were subjected to a low-intensity, run-to-exhaustion protocol on a motorized treadmill, as previously described (Fujita et al., 2018). Mice were familiarized with the treadmill (Muromachi Kikai, Tokyo, Japan) for 10 min at 10 m/min for 2 consecutive days. The following day, mice were run at 10 m/min for 30 min, 11 m/min for 15 min, and 12 m/min for 15 min. Finally, the speed was incrementally increased by 1 m/min every 10 min until the mouse exhibited exhaustion. The endpoint was reached when the mouse sat on the shock grid at the back of the treadmill for longer than 5 s.

### Electron Microscopy

Electron microscopic examinations were performed as previously described (Fujita et al., 2018). Muscle samples were fixed in 2.5% glutaraldehyde in 0.1M phosphate buffer (pH 7.4), for 4 h at 4°C. Post-fixation, the samples were incubated with 1% osmium tetroxide for 2 h at 4°C. Muscle samples were then dehydrated in a graded series of ethanol and embedded in Epon 812. Ultrathin sections were cut using an ultramicrotome (Ultracut S; Leica, Vienna, Austria) with a diamond knife and then stained with uranyl acetate and lead nitrate. Samples were visualized using an electron microscope (JEM-1200EX; Jeol, Tokyo, Japan). The number of altered mitochondria was determined in plantaris (PLA) muscles as previously described with minor modification (Paolini et al., 2015). Intermyofibrillar mitochondria with any one of the following ultrastructural alterations were defined as altered mitochondria: (a) swollen mitochondria (for PTG samples, with disruption of internal cristae), (b) mitochondria with clear disruption of the external membrane and/or internal cristae, (c) mitochondria containing vacuoles. Under electron microscopic observation, altered mitochondria were found to be non-uniformly scattered. Images were taken in fibers with altered mitochondria. The number of altered mitochondria was quantified in 200–1400 μm^2^ of representative electron-microscopic area per mouse. At least two fields per mouse were used for the quantification (*n* = 3–7 mice).

### Histological Assessment

Immunohistochemical analysis was performed as previously described (Seko et al., 2016). Muscle samples were frozen in isopentane, cooled with liquid nitrogen, and stored at −80°C until use. Frozen muscle cross-sections (8 μm thick) were fixed with 4% paraformaldehyde, blocked with a MOM kit (Vector Laboratories, Burlingame, CA, United States), and incubated with primary antibodies at 4°C overnight. Samples were visualized by using appropriate species-specific Alexa Fluor 488 and Alexa Fluor 568-conjugated secondary antibodies (Life Technologies, Carlsbad, CA, United States). The following primary antibodies were used: mouse anti-type IIa myosin heavy chain (MyHC) antibody (SC-71), mouse anti-type IIb MyHC antibody (BF-F3) [Deutsche Sammlung von Mikroorganismen (Braunschweig, Germany)]. When stained with anti-type I MyHC antibody (BA-D5), type I fibers were not observed in PLA (data not shown). Thus, type IIa/IIb-unstained fibers were defined as type IIx fibers. To visualize β-galactosidase staining, muscle sections were fixed with 4% paraformaldehyde for 3 min and then incubated in 5-bromo-4-chloro-3-indolyl-*b*-D-galactopyranoside (X-gal) solution overnight at 37°C, rinsed three times with distilled water, briefly air-dried, and then mounted on coverslips.

### Glucose- and Insulin- Tolerance Tests

A glucose-tolerance test (GTT) was performed by intraperitoneal glucose injection (1 g/kg body weight) after overnight food withdrawal (16 h). Blood-glucose concentrations were measured using Accu-Chek (Roche, Basel, Switzerland) before glucose injection (0 min) and 30, 60, and 120 min after glucose injection. An insulin-tolerance test (ITT) was performed by intraperitoneal insulin injection (1.0 U/kg body weight) after 6 h of fasting. Blood-glucose concentrations using Accu-Chek were measured before insulin injection (0 min) and 30, 60, 90, and 120 min after insulin injection.

### Gene Expression Analysis

Quantitative real-time PCR was performed to determine mRNA expression levels. Total RNA was extracted from muscle using Isogen II (Nippon Gene, Tokyo, Japan), according to the manufacturer’s instructions. RNA was reverse transcribed into cDNA using a ReverTra Ace kit with genomic DNA remover (Toyobo, Tokyo, Japan). Real-time PCR was performed with Thunderbird SYBR quantitative PCR mix (Toyobo, Tokyo, Japan) and CFX96 Touch real-time PCR detection system (Bio-Rad, Tokyo, Japan). The expression levels of selected genes were analyzed using standard curve method and the values were normalized against *TATA box binding protein* (*TBP*). Primer sequences were as follows: *TBP* [forward(F) 5′-CAGATGTGCGTCAGGCGTTC-3′ and reverse (R) 5′-TAGTGATGCTGGGCACTGCG-3′]; *Zmynd17* (F 5′-TAGGGCTTAACAGGCACTGGTCCCC-3′ and R 5′-TTCTTGTGCTTTCGCCGCCGTG-3′).

### Statistical Analysis

For statistical comparisons of two conditions, Student’s unpaired, two-tailed, *t*-test was performed. For comparisons between more than two groups, one-way or two-way analysis of variance (ANOVA) were performed according to the experimental design, followed by Bonferroni’s multiple comparison tests. Statistical analysis was performed in Microsoft Excel or using GraphPad Prism (version 8). For all statistical tests, *p* < 0.05 was regarded as statistically significant. All error bars represent means ± standard error of the mean (SEM), *n.s.* represents statistically not significant.

## Results

### *Zmynd17* Is Predominantly Expressed in Glycolytic Muscle

**FIGURE 1:**
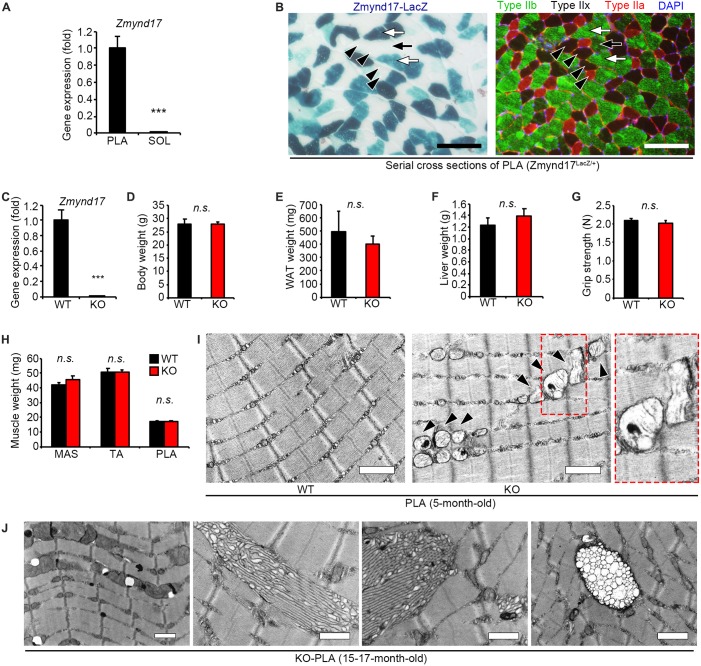
*Zmynd17* deletion causes morphologically alteration of mitochondria in glycolytic muscle. **(A)** qPCR analysis of *Zmynd17* mRNA expression in skeletal muscle (*n* = 5, ^∗∗∗^*p* < 0.001). **(B)** Representative images of cross-section of PLA stained with X-gal (left). Serial section was immunostained for MyHC IIb (green) and IIa (red) and DAPI (blue). Scale bar = 100 μm. White arrows show X-gal-positive type IIb fibers, and black arrows show X-gal-negative IIb fibers. Black arrowheads show type IIa and IIx fibers, both of which are X-gal-negative. **(C)** *Zmynd17* expression detected by qPCR (*n* = 5, ^∗∗∗^*p* < 0.001). **(D–H)** Assessment of *Zmynd17*-KO mice (*n* = 5). **(I)** Representative electron micrographs of PLA. Dotted square shows a magnified image. Arrowheads show altered mitochondria. Scale bar = 1 μm. **(J)** Electron micrographs of abnormal structures found in PLA of 15–17-month-old KO mice. All scale bars = 1 μm.

*Zmynd17* gene has been reported to be preferentially expressed in glycolytic muscle, but is expressed at notably lower levels in oxidative muscle and the other tissues, including the heart, liver, and kidney (Fujita et al., 2018). In the present study, we first confirmed that there is a significant difference in *Zmynd17* expression between soleus (SOL); oxidative muscle, and plantaris (PLA); glycolytic muscle (Figure 1A). β-Gal staining of PLA section revealed that *Zmynd17* expression is limited to type IIb fibers and is not seen in type IIa or IIx fibers (Figure 1B).

### *Zmynd17* Deletion Results in Abnormal Mitochondrial Morphology That Is Found to Be Accelerated With Aging

*Zmynd17*-KO mice have been reported to grow normally. However, they exhibit morphological alteration in mitochondria (Fujita et al., 2018). 5-month-old mice did not show any apparent differences in body weight, WAT (white adipose tissue) weight, liver weight, grip strength, and muscle weight (Figures 1C–H). However, when muscle tissues from *Zmynd17*-KO mice were visualized using electron microscopy, morphologically abnormal mitochondria with disrupted cristae with vacuoles or swollen shaped were observed. Such abnormalities were rarely found in WT muscle (Figure 1I). Mitochondrial quality and cellular senescence have been claimed to have strong relevance. At 15–17 months of age, morphological alteration of microstructures (tubular aggregates and multivesicular bodies) and organelles including mitochondria in *Zmynd17*-KO became even more obvious (Figure 1J). These results indicate that mitochondrial quality is abnormally regulated in *Zmynd17*-KO muscle.

### Voluntary Exercise Ameliorates Mitochondrial Abnormalities of *Zmynd17*-KO Mice

**FIGURE 2:**
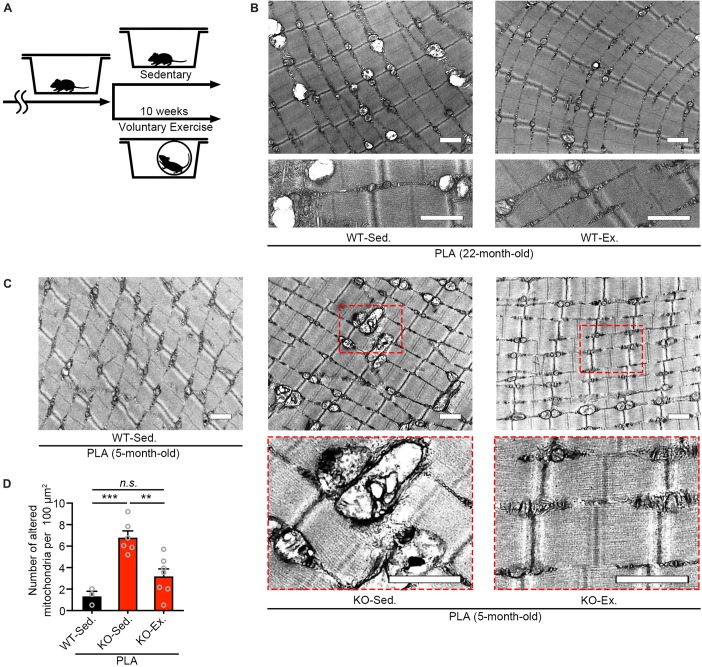
Voluntary exercise ameliorates mitochondrial abnormalities of Zmynd17-KO mice. **(A)** Experimental design. Mice were randomly divided into two groups. The voluntary exercise group was kept in a cage with a running wheel for 10 weeks before sampling. **(B,C)** Representative electron micrographs of PLA from each group. Dotted squares show magnified images. All scale bars = 1 μm. **(D)** Quantification of morphologically altered mitochondria. The number of altered mitochondria was quantified in 200–1400 μm^2^ of representative electron-microscopic area per mouse (WT-Sed., *n* = 3 mice; KO-Sed., *n* = 6 mice; KO-Ex., *n* = 7 mice; ^∗∗^*p* < 0.01, ^∗∗∗^*p* < 0.001).

Our previous study has shown that *Zmynd17*-KO mice exacerbates mitochondrial dysfunction when exposed to metabolic stress induced by a high-fat-diet or aging (Fujita et al., 2018). Numerous studies have reported that physical exercise improves mitochondrial dysfunction with aging (Radak et al., 2005; Safdar et al., 2011a; Cartee et al., 2016; Joseph et al., 2016). Consistent with these results, we found that 10 weeks of voluntary exercise reduced the accumulation of morphologically abnormal mitochondria in plantaris muscle of WT aged mice (Figures 2A,B). We further examined the effect of voluntary exercise on mitochondria in muscle lacking *Zmynd17* and found that voluntary exercise significantly ameliorates abnormal morphology of muscle mitochondria in *Zmynd17*-KO mice (Figures 2C,D). These results suggest that the beneficial effect of consecutive exercise on mitochondrial quality control is not mediated by the activity of Zmynd17.

### Overexpression of PGC1α Increases Abnormal Mitochondria Number in Muscle Lacking *Zmynd17*

**FIGURE 3:**
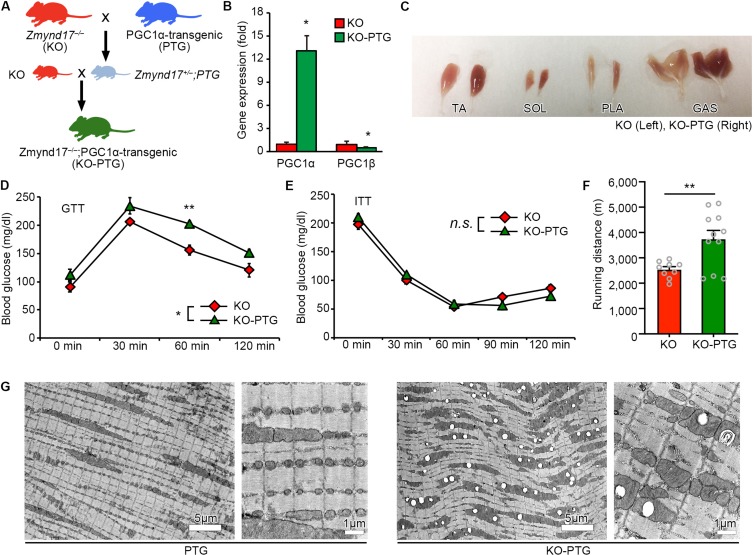
Overexpression of PGC1α in *Zmynd17*-KO mice causes apparent morphological alteration in mitochondria. **(A)** Schematic diagram of transgenic mice crossing. **(B)** qPCR analysis of PGC1α and PGC1β expression (KO, *n* = 3; KO-PTG, *n* = 4, ^∗^*p* < 0.05). **(C)** Representative images of muscles in KO and KO-PGC1α mice. **(D,E)** GTT and ITT were performed in KO and KO-PTG mice (KO, *n* = 4; KO-PTG, *n* = 6). The data was analyzed by Two-way Repeated Measures ANOVA (Source of variation; **(D)** KO vs. KO-PTG, ^∗^*p* < 0.05; time, *p* < 0.001; Interaction, *p* = 0.65. ^∗∗^*p* < 0.01; **(E)** KO vs. KO-PTG, *p* = 0.88; time, *p* < 0.001; Interaction, *p* = 0.87). **(F)** Running distance in KO and KO-PTG mice (KO, *n* = 9; KO-PTG, *n* = 11, ^∗∗^*p* < 0.01). **(G)** Representative electron micrographs of PLA from PTG mouse and KO-PTG mouse.

**FIGURE 4:**
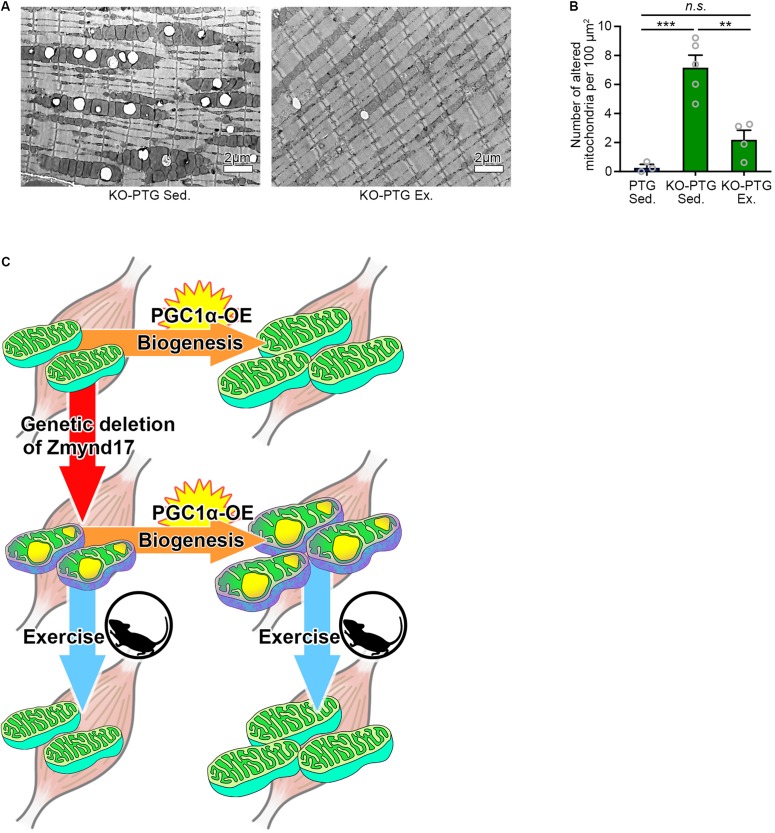
Voluntary exercise attenuates mitochondrial abnormalities of *Zmynd17-KO;PGC1*α*-transgenic* mice. **(A)** Representative electron micrographs of PLA from KO-PTG mice with or without 10 weeks of voluntary exercise. **(B)** Quantification of morphologically altered mitochondria in PLA. The number of altered mitochondria was quantified in 200–1400 μm^2^ of representative electron-microscopic area per mouse (PTG Sed., *n* = 3 mice; KO-PTG Sed., *n* = 5 mice; KO-PTG Ex., *n* = 4 mice, ^∗∗∗^*p* < 0.001). **(C)** A graphical summary of the study.

PGC1α is a nuclear receptor coactivator that promotes mitochondrial biogenesis and the oxidative metabolic program in muscle (Lin et al., 2002; Tadaishi et al., 2011; Senoo et al., 2015). As we have previously shown, metabolic stress exacerbated the systemic metabolism and mitochondrial morphological dysfunction in limb muscles of *Zmynd17*-KO mice (Fujita et al., 2018). We hypothesized that PGC1α attenuates mitochondrial abnormalities induced by Zmynd17 inactivation. To test this hypothesis, we crossed muscle-specific PGC1α overexpressing (PGC1α-Tg; PTG) mice with *Zmynd17*-KO mice to generate a *Zmynd17*-KO-PTG (KO-PTG) mouse line (Figure 3A). Introduction of the Pgc1α transgene markedly upregulated PGC1α expression in muscle of KO-PTG mice, but downregulated PGC1β levels (Figure 3B). KO-PTG mice exhibited a switch from glycolytic fibers to oxidative fibers (Figure 3C). To examine the impact of PGC1α overexpression on systemic metabolism in the presence or absence of Zmynd17, we performed GTT and ITT in mice that were fed a normal diet. KO-PTG mice exhibited significant glucose intolerance than Zmynd17-null mice (Figure 3D), while there was no significant difference in ITT between the two mice (Figure 3E). Treadmill running test showed that endurance exercise ability of Zmynd17-KO mice is improved by PGC1α overexpression (Figure 3F). We next examined mitochondrial morphology in KO-PTG muscle by electron microscopy. Overexpression of PGC1α resulted in an increase in mitochondrial number in muscle of PTG mice (Figure 3G). However, KO-PTG mice had structurally abnormal mitochondria with disrupted cristae that were abundant in the muscle (Figures 3G, 4B). These data suggest that Zmynd17 is required for maintaining mitochondrial integrity, which cannot be compensated by PGC1a expression in skeletal muscle.

### Voluntary Exercise Ameliorates Mitochondrial Abnormalities of Muscle in *Zmynd17-KO;PGC1*α*-Tg* Mice

Finally, we tested whether voluntary exercise can reduce the vacuole accumulation by mitochondria in KO-PTG mice. We found that voluntary exercise ameliorated mitochondrial abnormalities in KO-PTG mice (Figures 4A,B). These results further strengthen the idea that exercise could be an optimal therapeutic intervention strategy even in the existence of abnormal mitochondria induced by Zmynd17 dysfunction.

## Discussion

*Zmynd17*, also known as *Mss51*, is a muscle-specific gene in humans and mice (Moyer and Wagner, 2015). Our previous study has shown that *Zmynd17* deletion affects glucose metabolism in the whole body only when exposed to metabolic stress induced by intake of high-fat-diet or aging. Young *Zmynd17*-KO mice fed with normal-diet, however, do not exhibit any difference in GTT and ITT when compared to WT mice (Fujita et al., 2018). There are no significant differences in body weight, WAT weight, liver weight and grip strength in 5-month-old KO mice, while abnormal mitochondria can be observed in muscle (Fujita et al., 2018). Consistent with our previous findings, accumulation of altered mitochondria was remarkably increased in muscle of 15–17 months old middle-aged mice lacking *Zmynd17*. Thus, it is likely that the phenotypes observed in *Zmynd17*-KO mice develop in a lifestyle- or age-dependent manner. In addition to the altered mitochondria, we observed abnormal microstructures such as tubular aggregates and multivesicular bodies. These structures are also found in muscle of aged mice (Lin et al., 2018), suggesting that Zmynd17-deletion accelerates muscle senescence. It has been reported that type IIb fibers, but not IIx or IIa fibers, are markedly affected by aging, which is related to muscle atrophy, known as sarcopenia (Holloszy et al., 1991; Uchitomi et al., 2019). Presumably, type IIb fibers may be more susceptible to metabolic stress compared to slow-type fibers. It can be speculated that type IIb fibers highly express *Zmynd17*, which may play a compensatory role to protect mitochondria from metabolic stress induced damage in a glycolytic muscle-specific manner.

Mitochondrial functional decline contributes to aging, and thus, understanding how their quality is controlled is important. It has been shown that consecutive exercise prevents deleterious effects of aging by improving mitochondrial function in muscle (Cartee et al., 2016; Joseph et al., 2016; Safdar et al., 2016). In the present study, we showed that 10 weeks of voluntary exercise significantly reduced the number of morphologically abnormal mitochondria in muscles of both aged mice and *Zmynd17*-KO mice. These findings suggest that the beneficial effect of exercise on mitochondrial quality in muscle is, at least, independent of Zmynd17 activity. Because exercise impacts mitochondrial dynamics in multiple ways, including biogenesis, mitophagy, fusion, and fission, abnormal mitochondria induced by Zmynd17-dysfunction or aging could be eliminated by such mechanisms.

Muscle contractile activity upregulates PGC1α, a master regulator of mitochondrial biogenesis, through adenosine monophosphate (AMPK) and sirtuin 1 (Sirt1) (Irrcher et al., 2008; Cantó et al., 2010; Price et al., 2012; Menzies et al., 2013). One of the beneficial effects of exercise is believed to be upregulation of PGC1α (Pilegaard et al., 2003; Sandri et al., 2006; Handschin and Spiegelman, 2008; Price et al., 2012; Wrann et al., 2013) In the present study, we showed that PGC1α overexpression in muscle leads to development of dense mitochondria with normal cristae structure and increases endurance exercise capacity. Interestingly, in the absence of *Zmynd17*, PGC1α-overexpression did not ameliorate mitochondrial morphology, but instead increased the number of abnormal mitochondria in muscle. Muscle-specific overexpression of PGC-1α enhanced endurance capacity in *Zmynd17*-KO mice, which is probably due to a muscle fiber-type-switch to slow fibers and increased mitochondrial content. It is likely that PGC1α stimulates mitochondrial biogenesis but is unable to compensate for Zmynd17 function that regulates mitochondrial quality control in muscle. We found that voluntary exercise significantly reduces the number of abnormal mitochondria in KO-PTG mice. Thus, PGC1α-stimulated mitochondrial biogenesis and Zmynd17-mediated mitochondrial quality control seem to be distinct mechanisms (Figure 4C). This provides a novel aspect on mitochondrial biogenesis and quality control.

## Conclusion

We demonstrated that voluntary exercise ameliorates morphological abnormalities of limb muscle mitochondria in the absence of Zmynd17. Our findings shed light on a novel molecular mechanism of mitochondrial biogenesis and quality control in muscles and highlight the importance of developing exercise-based therapies for the treatment of metabolic diseases, such as type 2 diabetes.

## Data Availability Statement

The datasets generated for this study are available on request to the corresponding author.

## Ethics Statement

The animal study was reviewed and approved by the Animal Committee of Nagasaki University.

## Author Contributions

KY designed and performed the experiments, interpreted and analyzed the data, and wrote the manuscript. RF, DS, TS, and SM performed the experiments, and interpreted and analyzed the data. YO designed the experiments, interpreted the data, assembled the input data, and wrote the manuscript. All authors discussed the results and implications, and commented on the manuscript.

## Conflict of Interest

The authors declare that the research was conducted in the absence of any commercial or financial relationships that could be construed as a potential conflict of interest.
